# Maternal acceptability of pulse oximetry screening at home after home birth or very early discharge

**DOI:** 10.1007/s00431-017-2883-2

**Published:** 2017-03-09

**Authors:** Ilona C. Narayen, Adrian A. Kaptein, Janine A. Hogewoning, Nico A. Blom, Arjan B. te Pas

**Affiliations:** 1 Division of Neonatology, Willem-Alexander Children’s Hospital, Leiden University Medical Center, Postzone J6-S, room J6-217, Albinusdreef 2, 2333 RC Leiden, The Netherlands; 2 Department of Medical Psychology, Leiden University Medical Center, Leiden, The Netherlands; 3 Division of Pediatric Cardiology, Willem-Alexander Children’s Hospital, Leiden University Medical Center, Leiden, The Netherlands

**Keywords:** Acceptability, Questionnaire, Neonate, Screening, Pulse oximetry

## Abstract

The Netherlands has a unique perinatal healthcare system with a high rate of home births and very early discharge after delivery in hospital. Although we demonstrated that pulse oximetry (PO) screening for critical congenital heart disease is feasible in the Netherlands, it is unknown whether parents find the screening acceptable when performed in home birth setting. We assessed the acceptability of PO screening to mothers after screening in home setting. A questionnaire was sent electronically to mothers who gave birth and/or had postnatal care under supervision of a community midwife participating in the Pulse Oximetry Leiden Screening (POLS) study, a feasibility study of PO screening in the Dutch care system, performed in the Leiden region, the Netherlands. The questionnaire included questions based on satisfaction, general feelings, and perceptions of PO screening. A total of 1172/1521 (77%) mothers completed the questionnaire. Overall, mothers were happy with the performance of the test (95%), thought their baby was comfortable during the screening (90%) and did not feel stressed while the screening was performed (92%). Most mothers would recommend the test to others (93%) and considered the test important for all babies (93%).

*Conclusion*: Mothers of newborns participating in the study found the PO screening acceptable when performed at home.

**Table:** 

**What is Known**:
• *Pulse oximetry screening for critical congenital heart defects is (cost)effective and acceptable to mothers when performed in hospital*.
**What is New:**
• *Pulse oximetry screening for critical congenital heart defects is also acceptable for mothers when the screening is performed at home*.

## Introduction

Pulse oximetry (PO) is an accurate and cost-effective screening tool for critical congenital heart defects (CCHD) in newborns and has the advantage to detect other important neonatal pathology as secondary targets [1, 4, 8, 10]. However, PO screening has not been implemented in the Dutch universal screening program [2]. The Dutch perinatal health care system is unique, with a high rate of home births (18%) and very early discharge from hospital after uncomplicated deliveries (<5 h). Community midwives supervise 33% of all deliveries in the Netherlands, either at home or at a birthing facility or hospital [6]. Their first follow-up visit of mother and newborn is at day two or three of life (day of birth is day one). With an adapted protocol, the Pulse Oximetry Leiden Screening (POLS) study showed that the use of PO screening after home births and early hospital discharge is both safe and feasible and could be easily implemented in the daily routine of community midwives in the Leiden region in the Netherlands [3, 5].

The burden of a screening is an important factor to consider when implementing a new screening strategy [11]. PO screening in hospital settings was proven to be acceptable to both mothers and clinical staff [1, 7, 9]. However, taking into account the unique perinatal healthcare system in the Netherlands, it is unknown whether mothers find the screening also acceptable when performed at home. A positive screening at home leads to referral to a hospital, which can be highly uncomfortable and disruptive for the childbed of a newborn and for the mother, since it requires transfer in the first days (sometimes even hours) after delivery, while they are still recovering from the delivery. Furthermore, parents can experience stress and insecurity about the condition of the baby. Therefore, it is possible that performing the screening at home might be less acceptable for mothers when compared to screening in hospital.

We aimed to assess the acceptability of PO screening for the mothers participating in the POLS study in the Leiden region.

## Materials and methods

### Participants and procedures

The POLS study was performed between October 2013 and October 2014 in the Leiden region, the Netherlands. This prospective study was conducted in one academic hospital (Leiden University Medical Center (LUMC)), two regional hospitals (Rijnland Hospital Leiderdorp and Diaconessenhuis Leiden), and 14 regional community midwifery practices. PO measurements were performed pre- and post-ductally at two moments; at least 1 h after birth (median 1.8 h after birth) and at day two or three of life, at home during the first follow-up visit of the community midwife, or in hospital in case of prolonged hospital admission. The screening was abnormal in case of a pre- or post-ductal oxygen saturation below 90%, or with either a difference between the two limbs of >3%, and/or if the measurements at both limbs were <95% [3, 5].

In Dutch perinatal care, a community midwife is responsible for the postnatal care of a mother and newborn in the first 8–10 days following childbirth, when the mother and newborn are at home (after hospital discharge or in case of home birth). Mothers who gave birth and/or had postnatal care under supervision of a community midwife during the POLS study were invited by email by their midwife to complete a questionnaire online. This questionnaire consisted of selected and translated questions from the questionnaire for mothers that was used in the PulseOx study in the UK (Table 1) [1].

**Table 1:** Maternal perception on pulse oximetry screening

	Strongly agree^a^ Yes, definitely^b^, *n* (%)	Agree^a^ Yes, probably^b^, *n* (%)	Neither agree or disagree^a^ I do not know^b^, *n* (%)	Disagree^a^ Probably not^b^, *n* (%)	Strongly disagree^a^ Definitely not^b^, *n* (%)	Total, *n* (%)
Overall, I was happy with the way the test was done.^a^	523 (45)	585 (50)	45 (4)	16 (1)	3 (0.3)	1172 (100)
My baby was very comfortable when the test was done.^a^	536 (46)	513 (44)	82 (7)	36 (3)	5 (0.4)	1172 (100)
I did not feel stressed while the test was being done.^a^	591 (50)	491 (42)	56 (5)	31 (3)	3 (0.3)	1172 (100)
Do you think it was important for your baby to have the test?^b^	683 (58)	340 (29)	116 (10)	31 (3)	2 (0.2)	1172 (100)
Do you think it is important for all babies to have the test?^b^	781 (67)	306 (26)	76 (7)	8 (0.7)	1 (0.1)	1172 (100)
Would you recommend the test to someone else?^b^	804 (69)	286 (24)	72 (6)	9 (0.8)	1 (0.1)	1172 (100)

^a^Strongly agree–strongly disagree

^b^Yes, definitely–definitely not

### Outcome

The outcome of this study was maternal acceptability. The questions focused on maternal perceptions during the measurement of the PO screening (happiness with test; comfort of baby; perceived stress), the extent to which mothers would recommend the test to someone else and whether they thought the test was important for their or all babies. Higher scores implied more positive perceptions.

### Statistical analyses

Data are presented as numbers and percentages. Statistical analyses were performed with IBM SPSS Statistics version 23.0.

### Ethical considerations

The Medical Ethical Committee of the LUMC approved this study.

## Results

### Participation in questionnaire study

In the POLS study, 3059 babies were included of which in 1521 (50%) infants at least one screening was performed at home (908 (60%) both screenings, 613 (40%) only second screening). The mothers of the babies where screening was performed at home were invited to complete the questionnaire of which 1172/1521 (77%) mothers completed the questionnaire.

### Maternal acceptability

Table 1 shows the perceptions of mothers for the screening test. The majority of mothers were happy with how the test was performed (95%) and did not feel stressed during the test (92%). Most mothers (90%) thought that their babies were comfortable when the screening was performed. The majority of the mothers considered the test was important for the well-being of their own baby (87%) and for all (also other) babies (93%). The vast majority of mothers (93%) would recommend the test to someone else, while only 1% would not.

## Discussion

Since an adapted protocol was used in the POLS study to facilitate PO screening after home births and with early discharge in the Netherlands, the acceptability of mothers was assessed. The vast majority of mothers were satisfied with the screening; most mothers considered it important for their babies and other babies and would recommend the test to others. Based on these results, our study implicates that the implementation of PO screening at home would be acceptable for the mothers.

Acceptability for neonatal PO screening has been assessed before, although this was in different settings, after hospital deliveries [7, 9]. However, their findings are comparable to ours. In a large study in the UK, false positive results did not increase anxiety and mothers were overall satisfied with the PO test [1].

The general maternal acceptability in our study might be explained by several factors. First, it was not mandatory to test one’s baby and therefore participation after informed consent was a conscious and voluntary choice. For this reason, mothers were probably positively disposed towards the PO screening before participation. Other aspects of the test as being not time-consuming and noninvasive will also positively influence the acceptability. The PO screening is painless and not dangerous for the baby. There are no known risks, and the parents were informed about the safety of the measurement before screening. Furthermore, the measurement was performed by the mother’s own healthcare provider. The possibility of early detection of potential life-threatening pathology may also have influenced the acceptability due to the possibility of prompt treatment before deterioration.

There were some limitations in this study. For example, the decoded (anonymous) storage of data in order to guard the privacy of the mothers entering the online questionnaire made it impossible to link the test results to the participants. As a result, this study did not distinguish between mothers of newborns with false positive, true positive, and true negative screening. However, the numbers of false positives were low, and there were no true positives or false negatives in the POLS study, which makes it difficult to make a valid comparison between the true and false positive and negatives.

This study was conducted in the Leiden region, a middle-sized city in the urban agglomeration of Netherlands, and might therefore not be representative for the rest of the country, including the larger cities or rural areas.

In conclusion, PO screening at home was acceptable to mothers participating in the POLS study.

## Abbreviations

CCHD: Critical congenital heart defects
LUMC: Leiden University Medical Center
PO: Pulse oximetry
POLS: Pulse Oximetry Leiden Screening
